# Ruxolitinib-Associated Infections in Polycythemia Vera: Review of the Literature, Clinical Significance, and Recommendations

**DOI:** 10.3390/cancers12113132

**Published:** 2020-10-26

**Authors:** Parvis Sadjadian, Kai Wille, Martin Griesshammer

**Affiliations:** University Clinic for Hematology, Oncology, Hemostaseology and Palliative Care Johannes Wesling Medical Center Minden, UKRUB, University of Bochum, Hans-Nolte-Straße 1, D-32429 Minden, Germany; parvis.sadjadian@muehlenkreiskliniken.de (P.S.); kai.wille@muehlenkreiskliniken.de (K.W.)

**Keywords:** polycythemia vera, PV, ruxolitinib, infection

## Abstract

**Simple Summary:**

Polycythemia vera (PV) is a chronic blood disease characterized by elevated red blood cells and splenomegaly. About 98% of all PV patients harbor the JAK2 mutation. Ruxolitinib (RUX), a JAK1/JAK2 inhibitor, received approval as a second-line indication in PV patients who are resistant or intolerant to standard therapy hydroxyurea in both the United States (2014) and Europe (2015). In the studies relevant to approval, RUX achieved excellent PV control. Due to its mechanism of action, RUX also has immunosuppressive effects. As expected, an increased rate of infection was observed in clinical studies and in practical application. In this overview, we have compiled all previous literature references on RUX and infections in PV. However, apart from a few individual cases with special infections and an increased rate of zoster infections, there are no exceptional high infection problems. Recommendations are given on how infections in RUX treated PV patients can be avoided.

**Abstract:**

Ruxolitinib (RUX), a JAK1/JAK2 inhibitor, is approved for second-line therapy in patients with polycythemia vera (PV) who are resistant or intolerant to hydroxyurea. Due to the immunomodulatory and immunosuppressive effect of RUX, there is an increased susceptibility to infections. However, an increased risk of infection is inherent to even untreated myeloproliferative neoplasms (MPN). To obtain more information on the clinical significance of RUX-associated infections in PV, we reviewed the available literature. There is no evidence-based approach to managing infection risks. Most data on RUX-associated infections are available for MF. In all studies, the infection rates in the RUX and control groups were fairly similar, with the exception of infections with the varicella zoster virus (VZV). However, individual cases of bilateral toxoplasmosis retinitis, disseminated molluscum contagiosum, or a mycobacterium tuberculosis infection or a hepatitis B reactivation are reported. A careful assessment of the risk of infection for PV patients is required at the initial presentation and before the start of RUX. Screening for hepatitis B is recommended in all patients. The risk of RUX-associated infections is lower with PV than with MF, but compared to a normal population there is an increased risk of VZV infection. However, primary VZV prophylaxis for PV patients is not recommended, while secondary prophylaxis can be considered individually. As early treatment is most effective for VZV, patients should be properly informed and trained to seek medical advice immediately if cutaneous signs of VZV develop. Vaccination against influenza, herpes zoster, and pneumococci should be considered in all PV patients at risk of infection, especially if RUX treatment is planned. Current recommendations do not support adjusting or discontinuing JAK inhibition in MPN patients to reduce the risk of COVID-19.

## 1. Risk of Infections in Myeloproliferative Neoplasm (MPN)

Evidence that the risk of death from infections is increased in myeloproliferative neoplasms (MPN) is provided by information from Swedish registries, including 9285 patients with MPN and 35,769 matched controls [1]. In the MPN cohort, the 10-year probability of death from infection was 4.6% compared to 2.3% in controls. Within the various MPN subtypes, the corresponding 10-year mortality risk rate due to infection was 4.6% in polycythemia vera (PV), 2.5% in essential thrombocythemia (ET), and 10.4% in primary myelofibrosis (PMF). In PV, the most common cause of death was cardiovascular disease at 18.9%. Bacterial infections were responsible for the majority of infectious deaths. The authors concluded that an underlying MPN can lead to a higher susceptibility to infection, either because of the malignancy itself or because of long-term treatment of these chronic conditions. Interestingly, the risk of dying from infections decreased during the study period, likely due to better diagnostic and therapeutic options that have been developed over time.

Not so long ago, the same group assessed the risk of serious infections in MPN [2]. They performed a large population-based matched cohort study in Sweden including 8363 MPN patients and 32,405 controls. The risk of infection was significantly increased in all MPN subtypes, with 3.7 being the highest hazard ratio (HR) in myelofibrosis. The HR for both PV and ET was 1.7. Both bacterial and viral infections have been seen, but the greatest increase in risk was seen with sepsis with an HR of 2.6. However, this higher risk of infection with MPN did not differ significantly between treated (with hydroxyurea or interferon alpha) and untreated patients. The latter finding could indicate that the observed increased risk of infection in MPN is more inherent in the underlying malignancy.

Crodel et al. recently published the frequency of infections in an unselected group of 948 MPN patients as part of a multicenter patient-reported pilot study [3]. Within the past 12 months, more than half of all MPN patients (50.5%) reported one or more episodes of infection. The three most common infections were upper respiratory tract (35.7%), herpes virus (15.2%), and gastro-intestinal (14.2%) infections. Pneumonia (3.9%) and urinary tract infections (4.5%) were less common. Of all MPN included, myelofibrosis patients had the highest percentage of infectious events (57.4% ≥1 infection; *p* = 0.022). The corresponding rates in PV and ET were lower at 49.8% and 47.4%, respectively. The proportion of patients who reported at least one infectious episode during the last 12 months was highest in subjects treated with interferon alpha, the JAK1/2 inhibitor ruxolitinib (RUX), or combinations with RUX at 61.4%, 68.2%, and 69.8%, respectively. Patients receiving hydroxyurea or no medications had a lower risk of infection and the corresponding rates with ≥1 infectious episode were 36.9% and 55.6%, respectively. As previously reported, most herpes virus infections occurred in RUX treated patients either as RUX-monotherapy (25.3%) or with RUX-containing combinations (26.4%). Unexpectedly, the rate of herpes infections in patients treated with interferon alpha was similarly high at 22.8%. Only 11.4% of all MPN patients reporting on infections required hospitalization. Of note, hospitalization occurred more frequently in patients receiving RUX or a combination with RUX (*p* = 0.05).

All the data cited clearly indicate an increased risk of infection in MPN, which leads to higher mortality rates. This is especially true for patients with myelofibrosis. Treatment with RUX, in particular combinations with RUX, but also therapy with interferon alpha seem to be associated with an increased infection rate.

## 2. Therapy Management of Polycythemia Vera and the Current Role of Ruxolitinib Treatment

Patients with PV are at a higher risk of vascular and thrombotic complications and have a reduced quality of life due to a significant burden of symptoms [4]. The latter includes pruritus, fatigue, constitutional symptoms, microvascular disturbances, and bleeding. The cornerstone of PV treatment is phlebotomy along with low-dose acetylsalicylic acid. Further treatment decisions are based on risk stratification and, in the case of a high-risk situation, cytoreduction is indicated. High-risk factors include being over 60 years of age and previous arterial/venous thrombosis [5]. Other indications for starting cytoreductive therapy are symptomatic or progressive splenomegaly, severe disease-related symptoms, and platelet counts greater than 1500 × 10^9^ /L or leukocytosis >15 × 10^9^ /L [5]. According to the guidelines of the European Leukemia Net (ELN), first-line cytoreductive therapy for PV is started with either hydroxyurea or interferon alpha. However, the ELN experts agreed in their guideline that hydroxyurea should be used with caution in young patients. In February 2019, therapy with the novel ropeginterferon alfa-2b was approved by the European Commission as first-line therapy in adults for the treatment of PV without symptomatic splenomegaly.

Due to the crucial role of the dysregulated JAK-STAT pathway in the pathogenesis of MPN, which leads to an increased JAK-STAT signal and MPN associated inflammation, JAK inhibition strategies were used in clinical trials shortly after the discovery of the *JAK2 V617F* point mutation in 2005 [6]. About 60% of myelofibrosis and 98% of PV patients harbor the *JAK2* mutation (in PV either as *JAK2* exon 14 (95%) or *JAK2* exon 12 (3%) mutation). However, ruxolitinib is effective regardless of JAK2 mutation status given that all MPN patients have dysregulated JAK/STAT signaling. JAK1 is important for the signal transmission of pro-inflammatory cytokines, while JAK2 is the mediating receptor molecule for hematopoietic growth factors. In the last decade, JAK inhibitors have developed into the most interesting and promising class of active substances in MPN targeted therapy [7]. Following the positive results of the two central COMFORT trials [8,9], the Janus kinase *JAK1/JAK2* inhibitor ruxolitinib was approved in the United States in 2011 for the treatment of patients with intermediate-2 or high-risk primary or secondary MF. A year later, in 2012, RUX was also approved in Europe for patients with MF and splenomegaly and/or constitutional symptoms. Based on the results of the RESPONSE trials (RESPONSE and RESPONSE-2), ruxolitinib received approval as a second-line indication in PV patients who are resistant or intolerant to hydroxyurea in both the United States (2014) and Europe (2015) [10,11]. In both RESPONSE trials, ruxolitinib achieved control of both hematocrit and splenomegaly in these pre-treated PV cases with a median time of more than eight years since the initial diagnosis. In addition, the use of ruxolitinib led to a significant improvement in the burden of symptoms, as was determined using the MPN-SAF questionnaire.

However, in the RELIEF study, treatment with ruxolitinib was only associated with a trend toward improvement in total symptom score cytokine symptom cluster (TSS-C) [12]. The RELIEF (Randomized Switch Study from Hydroxyurea to Ruxolitinib for RELIEF of Polycythemia Vera Symptoms) trial was a randomized (1:1), multicenter, double-blind, double-dummy, phase III, and switch study to evaluate the efficacy and safety of ruxolitinib versus hydroxyurea to control disease-related symptoms in patients with PV who are currently reporting symptoms during HU monotherapy. Unexpectedly, a relatively high proportion of patients who received a stable hydroxyurea dose already reached the primary endpoint in RELIEF (29.6%). Other significant differences between PV patients in RELIEF and RESPONSE were a shorter median duration of PV (58.5 versus 98.4 months), a shorter median palpable spleen length (zero versus seven centimeters), and a lower mean blood count.

The MAJIC study is a phase II academic randomized trial of RUX versus best available therapy (BAT) in patients with PV and ET who were resistant or intolerant to hydroxyurea [13]. In the RUX arm, clinically and statistically significant improvements in complete and partial hematological responses were seen within one year compared to PV patients treated with BAT. From a molecular point of view, RUX treatment resulted in an increased rate of reduction in allele burden with 29.4% compared with 17.9% in the BAT arm. 

Today, RUX is indicated as a second-line therapy in PV patients who are resistant or intolerant to hydroxyurea (HU). In patients treated with hydroxyurea (HU), approximately 1 in 4 will develop resistance (≈11%) or intolerance (13%) to the drug. Specific criteria of HU intolerance/resistance have been proposed by the European Leukemia Net (ELN) Working Group [14]. These are after three months of ≥2 g/day of HU, any one of: (a) need for phlebotomy to keep hematocrit <45%; (b) uncontrolled myeloproliferation (i.e., platelet count >400 × 10^9^/L, and white blood cell count >10 × 10^9^/L); (c) failure to reduce massive splenomegaly by >50% by palpation or resolve splenomegaly-related symptoms. Or, at the lowest dose of HU required to achieve a complete or partial response, any one of: (a) absolute neutrophil count <1.0 × 10^9^/L; (b) platelet count <100 × 10^9^/L; (c) hemoglobin <100 g/L. Or, at any dose of HU presence of leg ulcers or other unacceptable HU related nonhematologic toxicities (e.g., mucocutaneous manifestations, gastrointestinal symptoms, pneumonitis, or fever).

## 3. Mechanism of the Immunosuppressive Effect of JAK Inhibition

All four members of the JAK family play key roles in regulating functions of lympho-hematopoietic cells such as proliferation, differentiation, and survival via cytokines and interferons [15]. Many cytokine receptors depend on Janus kinases for receptor phosphorylation such as the IFNγ receptor, the IL-6 receptor, and other γ- chain-containing receptors (such as IL-2 and IL-7) [16]. Hence, the JAK-STAT signaling pathway influences T cell development and proliferation [17]. 

**Figure 1:**
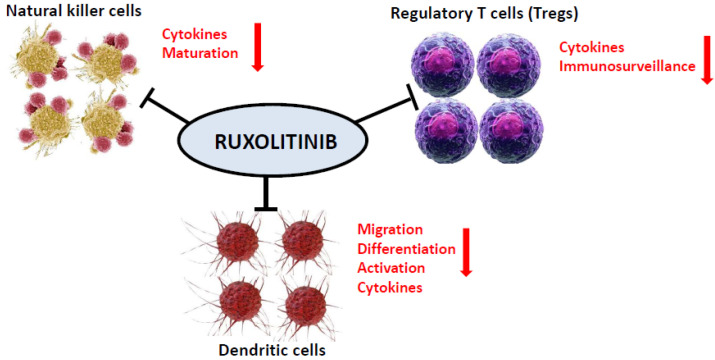
Ruxolitinib as a potent and selective inhibitor of both JAK1 and JAK2 protein kinases affects various components of both the innate and adaptive immune system such as dendritic cells, natural killer cells, T helper, and regulatory T cells.

As a potent and selective inhibitor of JAK1 and JAK2 protein kinases, ruxolitinib influences various components of the innate and adaptive immune system such as dendritic cells, natural killer cells, T helper, and regulatory T cells [18,19], resulting in a pronounced immunosuppressive effect (Figure 1).

### 3.1. Dendritic Cells (DC)

DCs are important antigen-presenting cells (APC) and phagocytic cells; after ingesting a pathogen, they migrate to a lymph node where they prime T lymphocytes. Once activated, DCs secrete numerous cytokines that induce the differentiation of naïve T cells into helper and effector T cells as well as regulatory T cells [20].

Ruxolitinib showed a dose-dependent inhibitory effect on the formation of dendritic cells (DCs) from monocytes [21]. The drug also inhibited DC activation, tissue migration, and the induction of allogeneic or antigen-specific T cell responses, including in vivo virus clearance [22]. 

### 3.2. Natural Killer (NK) Cells

NK cells are the main effector cells of the innate immune system, which are able to detect and carry out lytic activity on virus-infected cells or on malignant cells, and are therefore also important components of the immune competence and cancer immune surveillance mechanisms. NKs also produce cytokines, mainly IFN-γ and TNF-α, which modulate the differentiation of cells involved in adaptive immunity and induce the maturation of DCs [20].

In a trial with 28 MPN patients with or without ruxolitinib treatment and 24 healthy individuals, the effects of JAK inhibition on human NK cells were compared [23]. The authors found a decrease in NK counts in ruxolitinib-treated patients related to the incidence of clinically relevant infections, likely due to impaired NK cell maturation.

### 3.3. T Regulatory (Treg) Cells

CD4+ regulatory T cells control innate and adaptive immune responses and play an important role in the fight against viral, fungal, and protozoal infections. They also produce inhibitory cytokines such as IL-10 and TGF-β, which influence the function of T effector lymphocytes [20].

In a study involving 18 patients with myelofibrosis, treatment with ruxolitinib resulted in a long-lasting and irreversible reduction in circulating Tregs [24]. Keohane et al. showed in a cohort of 50 MPN patients that T regulatory cells are reduced in MPN patients compared to healthy controls and that this decrease is even more pronounced after JAK inhibitor therapy [25]. In addition, the pro-inflammatory cytokine production of Tregs was blocked and T helper cells (Th17) were functionally silenced, which is a possible explanation for the increased rate of atypical infections using JAK inhibitors. 

Due to the immunosuppressive effects of ruxolitinib, it was also approved for graft-versus-host disease (GvHD) and has also recently been studied in critically ill COVID-19 patients [26,27].

## 4. Infections in MPN Related to Ruxolitinib Treatment with a Special Focus on Polycythemia Vera

### 4.1. Infections Associated with Ruxolitinib in the COMFORT-I, COMFORT-II, and the JUMP Studies and in a Retrospective Analysis in Myelofibrosis 

Most of the data on RUX-associated infections in MPN are available for MF. This is due, on the one hand, to the fact that MF patients per se have higher infection rates, and on the other hand, more long-term data on the safety of RUX are retrievable. After three-years of follow-up of the patients in the COMFORT-I trial, urinary tract infections (10.5% in 0 to <12 months, 6.7% in 12 to <24 months, 7.7% in 24 to <36 months, and 6.0% after ≥36 months) and zoster infections (2.1% in 0 to <12 months, 3.5% in 12 to <24 months, 3.4% in 24 to <36 months, and 0% after ≥36 months) were the most common infections that occurred during randomized treatment with RUX [10,28]. It is important to note that long-term therapy with RUX has not seen an increase in the incidence of infections. In the period between the 12th and 24th month and between the 24th and 36th month, only two urinary tract infections with a grade of ≥III occurred. No other opportunistic infections occurred during long-term RUX therapy [28]. Long-term results from COMFORT-II provided more detailed information on infections of particular interest in patients treated with RUX. These included urinary tract infections (24.6%), pneumonia (13.1%), herpes zoster infections (11.5%), sepsis and septic shock (7.9%), and tuberculosis (1.0%) [29].

The JUMP expanded-access trial is the largest MF study to date and included 1144 RUX treated cases with intermediate and high-risk MF [30]. All-grade infections that occurred in ≥1% of MF patients included nasopharyngitis (6.3%), urinary tract infection (6.0%), pneumonia (5.3%), bronchitis (4.2%), herpes zoster (3.6%), influenza (3.0%), upper respiratory tract infection (2.9%), cystitis (2.5%), gastroenteritis (1.8%), respiratory tract infection (1.8%), and oral herpes (1.6%). Other infections included tuberculosis in three patients (0.3%) and Legionella pneumonia in one patient (0.1%). No hepatitis B reactivation has been reported.

In a retrospective analysis of 507 MF patients, a total of 112 patients (22%) had 160 infectious events (grade III–IV, 45%) with an incidence rate of 3.9% per patient per year. Most infections were bacterial (78%) and mainly affected the respiratory tract. The infection rate was significantly higher in 128 patients treated with ruxolitinib compared to the remaining 379 patients who did not have ruxolitinib (44% vs. 20%, *p* < 0.001) [31]. 

### 4.2. Infections Associated with Ruxolitinib in the RESPONSE and RELIEF Phase III Trials in Polycythemia Vera

In the RESPONSE trial, which included hydroxyurea resistant or intolerant PV patients with a spleen volume >450 cm^3^, the overall infection rate of each grade was 41.8% in the ruxolitinib group and 36.9% in the best available therapy group [10]. The rates of grade III or IV infections were 3.6% and 2.7%, respectively. Herpes zoster infections, all of grade I or II, occurred in seven patients in the ruxolitinib group (6.4%) compared with no patients on the standard therapy.

In the 80-week follow-up analysis from the RESPONSE trial, the rate of all infections (per 100 patient-years) was 29.4 (grade III and IV 4.0) in the ruxolitinib arm compared with 58.4 (grade III and IV 4.1) in the best available therapy group. The rate of herpes zoster (per 100 patient-years) in the ruxolitinib arm (all grades 5.3, grade III and IV 0.9) was still higher compared with the best available therapy group in which no herpes zoster infection occurred [32].

In the last update of this trial (five-year follow-up data), the infection rates in ruxolitinib-treated PV patients were still lower (per 100 patient-year of exposure: 18.9 in the ruxolitinib arm and 19.1 in the crossover population) compared to those in the best available therapy group (59.8 per 100 patient-year) [33]. However, herpes zoster infections were still more common in ruxolitinib-treated PV patients.

RESPONSE-2, an open-label, randomized (1:1) phase IIIb study compared the efficacy and safety of ruxolitinib with the best available therapy in patients with hydroxyurea resistant or intolerant PV who have a non-palpable spleen and thus were not eligible for the RESPONSE trial [11]. Upper respiratory tract infection rates, all grade I and II, were higher in the best available therapy group (9%) than in the ruxolitinib group (3%). Influenza and bronchitis (grade III or IV) occurred in two of 74 RUX patients (3%) compared to one of 75 patients (1%) in the best available therapy group. Herpes zoster infection (grade I–II) occurred in one of 74 RUX patients (1%) but was not reported in the best available therapy group. Only a single bacterial pneumonia (grade III) was observed and occurred in the best available therapy group.

At the three-year follow-up of the RESPONSE-2 trial, in the ruxolitinib arm, the rates of all grade and grade III/IV infections per 100 patient-years of exposure were 24.9 and 2.3, versus 33.7 and 3.7 in those receiving the best available therapy [34]. Again, the herpes zoster infection rates were higher in patients receiving ruxolitinib. All grade exposure-adjusted rates of herpes zoster infection (per 100 patient-years of exposure) were 3.8 in patients originally randomized to ruxolitinib, 7.5 in patients receiving ruxolitinib after crossover, and zero in the BAT arm. 

The safety profile of ruxolitinib observed in the RELIEF study was comparable to the data reported above from the RESPONSE and RESPONSE 2 studies [12]. During blinded treatment, there was only one herpes zoster infection in the ruxolitinib arm. No patient in the RELIEF study received prophylactic treatment for herpes zoster infection before or during the trial.

### 4.3. Ruxolitinib-Associated Infections Reported in Literature Reviews or Case Reports in MPN

There is a systematic review and meta-analysis in the literature with the primary aim of evaluating the incidence and severity of infectious complications in MPN patients treated with ruxolitinib [35]. A total of five randomized clinical phase III trials (COMFORT-I and -II, RESPONSE and RESPONSE-2, RELIEF), six phase IV studies (all MF patients), and 28 case reports with 31 patients were included [36,37,38,39,40,41,42,43,44,45,46,47,48,49,50,51,52,53,54,55,56,57,58,59,60,61,62,63]. In all three phase III PV trials (RESPONSE and RESPONSE-2, RELIEF), ruxolitinib was associated with a significantly increased risk of herpes zoster infection compared to the control group with 9/238 events in the ruxolitinib arm and 0/243 events in the control arm. In both RESPONSE studies, the odds ratios (ruxolitinib versus control) for any grade of infection or grade III/IV infection were slightly increased with ruxolitinib (1.11 (95%CI, 0.71, 1.73) and 1.54 (95% CI, 0.43, 5.57), respectively). Data on serious pneumonia were reported in three phase III studies (COMFORT-I and -II, RESPONSE-2), but ruxolitinib did not increase the risk of this complication compared with the placebo or best available therapy. No increased risk was calculated for staphylococcal infection or fatal sepsis. A total of 28 case reports with 31 patients with ruxolitinib-associated infections were also summarized in this article. However, only two of the 31 patients had a diagnosis of PV and another case was a PV with myelofibrosis [41,45,46]. The reported infections in these three cases were bilateral toxoplasmosis retinitis, disseminated molluscum contagiosum, and hepatitis B reactivation. All of these cases have already been published individually [36,37,38,39,40,41,42,43,44,45,46,47,48,49,50,51,52,53,54,55,56,57,58,59,60,61,62,63]. The most common infections in the remaining cases (MF (*n* = 8), post-PV MF (*n* = 8), post-ET MF (*n* = 2), PMF (*n* = 9), and MDS (*n* = 1)) were: tuberculosis (*n* = 10), hepatitis B reactivation (*n* = 4), and *Pneumocystis jirovecii* infection (*n* = 2). Four of the 10 patients (40%) with tuberculosis died. The median time from starting ruxolitinib treatment to infection was four and a half months (range, 1 to 51 months). Although the data published in this review suggest that the ruxolitinib-associated risk of infection may be clinically relevant, the authors concluded that the evidence is not solid enough to accurately estimate the risk of infection in ruxolitinib-treated patients [35].

In another review of the literature that describes 32 opportunistic infections in patients undergoing therapy with ruxolitinib, there is a large overlap with the review by Lussana et al. [64]. Most of the cases had MF (25/32), three were PV cases (the same as in Lussana et al. [35]), two patients had both MF and PV (the latter referred to as post-PV MF in Lussana et al. [35]), and the other two had cutaneous T-cell lymphoma and MDS (as in Lussana et al. [35]).

In a retrospective analysis based on the French pharmacovigilance database, 30 cases of infectious events related to ruxolitinib including opportunistic infections were reported in 26 patients [65]. The underlying diseases treated with ruxolitinib were PMF (*n* = 5), secondary myelofibrosis (*n* = 7), unspecified myelofibrosis (*n* = 8), polycythemia vera (*n* = 3), atypical myeloproliferative neoplasm (*n* = 1), and a graft versus host disease (*n* = 2). The reported infections were bacterial (*n* = 9), mycobacterial (*n* = 5), viral (*n* = 10), fungal (*n* = 4), protozoan (*n* = 1), and non-specified opportunistic infection (*n* = 1). The most frequently identified pathogen was the zoster virus (20%). Five cases of sepsis (16.6%) and a total of six deaths were reported. The causes for the deaths were sepsis (*n* = 3), multivisceral failure (*n* = 2), and one respiratory failure.

In a retrospective pharmacovigilance review, the number of patients who developed typical mycobacterium tuberculosis (MTB) and atypical mycobacterial infections (AMI) was evaluated while on treatment with ruxolitinib by utilizing the United States Food and Drug Administration adverse events reporting system (FAERS) [66]. In total, 91 reported cases of MTB associated with ruxolitinib were found compared with 4575 cases from all other drugs. The reporting odds ratio was significant at 9.2 (95% CI, 7.5 to 11.4). For AMI, 23 cases of AMI were identified with ruxolitinib compared to 1287 cases reported for all other drugs. The reporting odds ratio was significant at 8.3 (95% CI, 5.5 to 12.6). Twelve (13.2%) patients with MTB and eight (34.8%) with AMI died. The authors concluded that clinicians should be aware of this risk and consider evaluating patients for latent MTB and AMI prior to initiating treatment with ruxolitinib.

Further evidence for this ruxolitinib-associated risk for MTB and AMI comes from a recent retrospective analysis by a French center of 65 patients with PV or symptomatic primary or secondary myelofibrosis [67]. Two patients (one PMF and one post-PV MF, 2/65 = 3%) each developed one case of *Mycobacterium tuberculosis* and one case of *Mycobacterium avium* complex.

An overview of case reports of rare RUX-associated infectious complications is presented in Table 1.

## 5. Ruxolitinib and COVID-19

Overwhelming inflammatory responses contribute to shortness of breath in patients with COVID-19. As a JAK1/JAK2 inhibitor, ruxolitinib has pronounced anti-inflammatory properties and could, therefore, be an effective therapy for COVID-19 [68]. In a prospective study of 34 aged and high-risk comorbidity patients with severe COVID-19 infection, ruxolitinib was safe and was associated with significant clinical improvement, especially in lung function [27].

During the COVID-19 pandemic, the GIMEMA group (Gruppo Italiano Malattie Ematologico Maligne dell’Adulto) conducted a survey of 34 Italian centers to study the prevalence of infections in MPN [69]. A total of 1095 patients were treated with ruxolitinib, 829 for MF (75.7%) and 266 for PV (24.3%), and finally 36 were found positive for COVID-19. Of the 36 COVID-19 MPN patients 13 (36%) were asymptomatic, 13 had flu-like symptoms (36%), and ten were affected by pneumonia caused by COVID-19 (27.8%). Four positive patients required invasive and two non-invasive mechanical ventilation. Eight COVID-19 positive patients died with a death rate of 22%. As a result of this survey, it was found that the incidence of COVID-19 infection in MPN patients is rather low and a certain protective function of ruxolitinib cannot be ruled out.

Current recommendations do not recommend adjusting or discontinuing JAK inhibition in MPN patients to reduce the risk of COVID-19. On the contrary, stopping ruxolitinib in the event of COVID19 infection may be harmful and should be avoided if clinically feasible (www.hematology.org >COVID-19 and Myeloproliferative Neoplasms (ASH, Version 4.0; last updated September 17, 2020)).

## 6. Summary and Recommendations for Avoiding Complications from Ruxolitinib-Associated Infections with a Special Focus on Polycythemia Vera

Although the risk of ruxolitinib-associated infections is lower with polycythemia vera compared to myelofibrosis, there is an increased risk of infection. The most important infections that occur during ruxolitinib treatment are particularly herpes zoster infection, but bacterial (urinary tract infections, pneumonia, and sepsis) and other viral infections (such as influenza and hepatitis B reactivation) also occur. A careful assessment of the risk of PV patients is required at the initial presentation and before ruxolitinib is launched. It is important to note that there are no specific recommendations in the literature for the prevention of ruxolitinib-associated infections in PV. The existing literature is limited to general recommendations for the use of ruxolitinib. In a position paper of the European Conference on Infections in Leukemia (ECIL), Maschmeyer et al. recommended screening for HBV in all patients prior to initiating treatment with ruxolitinib [70]. Hepatitis B virus screening should include HBsAg, anti-HBs, and anti-HBc. In HBsAg-positive and/or anti-HBc-positive patients, prophylaxis preferably with entecavir is suggested. If anti-HBc is positive, HBV-DNA should be determined [70]. The European Society for Clinical Microbiology and Infectious Diseases (ESCMID) has also published in its consensus document that screening for chronic HBV infection should be performed prior to initiating treatment with JAK inhibitors [71]. To prevent HBV reactivation, the ESCMID group also suggests antiviral prophylaxis during therapy in HBsAg-positive patients. In addition, monitoring of HBV virus load in anti-HBc positive, HBsAg negative patients may be indicated to assess the possible reactivation of occult HBV infection. In the latter situation in particular, referral to a specialist should be considered in order to decide on prophylaxis and the continuation of ruxolitinib. Patients negative for HbsAg, anti-HBs, and anti-HBc should be considered for immunization [72]. Although recommended by some authors [73], there is no consensus in favor of routine testing for HCV and HIV before patients are started on ruxolitinib.

Due to the immunomodulatory and immunosuppressive effects of ruxolitinib, the occurrence of tuberculosis is a rare but special problem. Although the incidence is higher in MF patients treated with ruxolitinib, a precise medical history should always be taken before starting RUX treatment in PV, which takes into account individual risk factors such as risk groups or travel to endemic areas [70,71]. If there is a suspected case a Tuberculin Skin Test (TST) or (preferably) IFN-γ Release Assay, IGRA (i.e., Quanti-FERON test) should be performed [70].

We recommend vaccinating all PV patients over 60 years of age with a planned ruxolitinib treatment in accordance with the recommendations of the German Standing Committee on Vaccinations (STIKO) for “normal” people >60 years against influenza, herpes zoster, and pneumococci [3,74]. In addition, vaccination for meningococcal infection is (according to STIKO) suggested for those with an additional pre-existing comorbidity of the immune system [74]. Given the high reactivation rate of VZV under ruxolitinib, vaccination against VZV appears to be appropriate, with an inactivated vaccine being preferable to a live attenuated vaccine. There is not enough evidence to suggest that primary prophylaxis against the varicella zoster virus is mandatory (VZV) [72]. In the event of a history of severe or recurrent VZV infection, secondary prophylaxis can be carried out individually. In particular, patients should be thoroughly informed and educated in order to seek medical attention immediately if signs of cutaneous herpes zoster appear [72]. Early treatment with VZV antiviral drugs is most effective when started within the first three days of the rash onset. Recommendations for avoiding complications from ruxolitinib-associated infections in polycythemia vera (PV) are provided in Table 2.

## 7. Conclusions

Apart from an increased rate of VZV infections, the overall risk of infection appears to be lower for PV than for MF. However, there are currently no fundamentally different recommendations for PV to prevent complications from RUX-associated infections compared to MF. Whether this will also be the case in the future can only be answered based on long-term data from many PV patients treated with RUX. Long-term studies and corresponding registers on this topic are therefore essential.

## Figures and Tables

**Table 1:** Case reports of rare ruxolitinib-associated infectious complications.

Infectious Complication	MPN Subtype	References
Tuberculosis	PMF	[38,39,42,44,51,52,58,60]
Hepatitis B	PMF	[36,53]
	post-ET myelofibrosis	[62]
	PV	[46]
Cryptococcal meningoencephalitis	PMF	[37,64]
VZV meningoencephalitis	PV	[40]
Toxoplasmosis retinitis	PV	[41]
Pulmonary cryptococcosis	post-PV myelofibrosis	[57]
	PMF	[43]
Molluscum contagiosum	PV	[45]
*Pneumocystis jirovecii* pneumonia	PMF	[47,63,64]
EBV gastric ulcer	PMF	[48]
*Klebsiella* pneumonia liver abscess	PMF	[49]
Herpes simplex reactivation	MDS	[54]
Cytomegalovirus retinitis	PMF	[55]
Progressive multifocal leukoencephalopathy	PMF	[56]
*Talaromyces marneffei* infection	PMF	[59]
EBV driven lymphoproliferative disorder	post-PV myelofibrosis	[61]

**Table 2:** Recommendations for avoiding complications from ruxolitinib-associated infections in polycythemia vera (PV).

Recommended Actions/Tests	In Detail
Accurate history taking at the start and physical examination	Identify risk groups for infections like tuberculosis, identify other reasons for immunosuppression, and identify co-medication [70,71].
Baseline virology	Hepatitis B [70,71]. No consensus in favor of routine testing for HCV and HIV.
Hepatitis B screening	Includes HBsAg, anti-HBs, and anti-HBc [70]. If anti-HBc is positive, determine HBV-DNA [72].
Test in hepatitis B virus carriers	Close monitoring of liver function and plasma HBV-DNA level. Referral to a specialist [70].
Test, if tuberculosis is suspected	Tuberculin Skin Test (TST) or (preferably) IFN-γ Release Assay, IGRA (i.e., Quanti-FERON test) [70,72].
Vaccination	Age-appropriate vaccination against influenza, herpes zoster, and pneumococci [3,74]. Vaccination against meningococcal infection in patients with pre-existing comorbidity or the immune system *.
Varicella zoster virus (VZV)	No primary but secondary prophylaxis in individual cases. Inform and educate patients to seek immediate medical attention if signs of cutaneous herpes zoster develop [72]. VZV reactivation: consider vaccination against VZV with inactivated vaccine.

* According to STIKO, vaccination for meningococcal infection is suggested for those with an additional pre-existing comorbidity of the immune system [74].
